# Synthesis of Spinel Ferrite MFe_2_O_4_ (M = Co, Cu, Mn, and Zn) for Persulfate Activation to Remove Aqueous Organics: Effects of M-Site Metal and Synthetic Method

**DOI:** 10.3389/fchem.2020.00177

**Published:** 2020-03-24

**Authors:** Guang Xian, Shengyan Kong, Qiangang Li, Guangming Zhang, Ningyu Zhou, Hongbiao Du, Lijun Niu

**Affiliations:** ^1^School of Environment & Natural Resource, Renmin University of China, Beijing, China; ^2^Department of Military Installations, Army Logistics University of PLA, Chongqing, China; ^3^School of Energy & Environmental Engineering, Hebei University of Technology, Tianjin, China

**Keywords:** ferrite, M-site metal, synthesis, persulfate, organics

## Abstract

Metal species and synthetic method determine the characteristics of spinel ferrite MFe_2_O_4_. Herein, a series of MFe_2_O_4_ (M = Co, Cu, Mn, Zn) were synthesized to investigate the effect of M-site metal on persulfate activation for the removal of organics from aqueous solution. Results showed that M-site metal of MFe_2_O_4_ significantly influenced the catalytic persulfate oxidation of organics. The efficiency of the removal of organics using different MFe_2_O_4_ + persulfate systems followed the order of CuFe_2_O_4_ > CoFe_2_O_4_ > MnFe_2_O_4_ > ZnFe_2_O_4_. Temperature-programmed oxidation and cyclic voltammetry analyses indicated that M-site metal affected the catalyst reducibility, reversibility of M^2+^/M^3+^ redox couple, and electron transfer, and the strengths of these capacities were consistent with the catalytic performance. Besides, it was found that surface hydroxyl group was not the main factor affecting the reactivity of MFe_2_O_4_ in persulfate solution. Moreover, synthetic methods (sol–gel, solvothermal, and coprecipitation) for MFe_2_O_4_ were further compared. Characterization showed that sol–gel induced good purity, porous structure, large surface area, and favorable element chemical states for ferrite. Consequently, the as-synthesized CuFe_2_O_4_ showed better catalytic performance in the removal of organics (96.8% for acid orange 7 and 62.7% for diclofenac) along with good reusability compared with those obtained by solvothermal and coprecipitation routes. This work provides a deeper understanding of spinel ferrite MFe_2_O_4_ synthesis and persulfate activation.

**Graphical Abstract:**
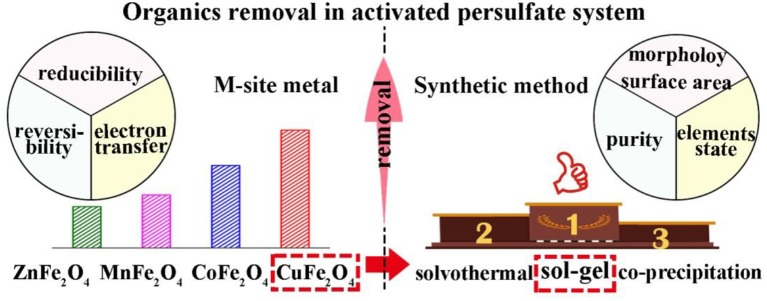
Effects of M-site metal and synthetic method of spinel ferrite on persulfate activation for organics removal.

## Highlights

- Effects of M-site metal and synthetic method on ferrite were investigated.
- Suitable ferrite and its synthetic method for PS activation were screened out.
- Catalytic PS performance was CuFe_2_O_4_ > CoFe_2_O_4_ > MnFe_2_O_4_ > ZnFe_2_O_4_.
- M-site metal affected MFe_2_O_4_ reducibility, M^2+^/M^3+^ reversibility, and electron transfer.
- Sol–gel method was ideal to synthesize ferrite to activate PS for organics removal.

## Introduction

Advanced oxidation processes (AOPs) that involve highly reactive radicals are powerful treatment techniques for the removal of organics in water, especially for removing highly toxic, persistent, and nonbiodegradable organics (Wang and Wang, 2018; Malvestiti et al., 2019). Among AOPs, activated persulfate (PS) process has received extensive attention. Compared with hydroxyl radical generated in conventional AOPs (Fenton or Fenton-like), sulfate radical ($\text{S}{{\text{O}}_{\text{4}}}^{•-}$) generated from PS has comparable oxidizing power (E^0^ = 2.5-3.1 V), higher selectivity (for benzene ring and unsaturated bond), longer half-life (30-40 μs), and greater stability, and is less influenced by natural organic materials (Oh et al., 2016; Alexopoulou et al., 2019). Moreover, PS offers some advantages over other AOP oxidants [e.g., H_2_O_2_ and peroxymonosulfate (PMS)], such as the ease of storage, high stability, high redox potential, good solubility, and relatively low cost (Xu and Li, 2010; Wacławek et al., 2017). Therefore, activated PS process is expected to be promising for treating organics.

Heterogeneous catalysis is the most studied method for PS activation, not only because of the energy conservation and ease of operation (vs. thermolysis, photolysis, radiolysis, etc.) (Oh et al., 2016; Zhu et al., 2019) but also owing to the mild reaction conditions, retrievability, and little metal dissolution (vs. homogeneous catalysis) (Wang and Wang, 2018). Available and efficient catalytic material is the priority in heterogeneous catalysis. Over the past decades, iron oxides have been generally used as heterogeneous catalysts because of their low price, abundant reserves, and nontoxicity (Li et al., 2017; Silveira et al., 2018). However, their weak catalytic activity limits the efficiency of pollutant removal (Lei et al., 2015). Hitherto, multimetallic iron-based materials can relieve this problem and render catalytic processes more efficient toward long-term application (Deng et al., 2017; Wacławek et al., 2017). With ongoing explorations, a typical bimetallic iron-based oxide, spinel ferrite with the general formula of MFe_2_O_4_ (M is a divalent 3d transition metal such as Co, Cu, Mn, and Zn), has attracted much attention (Lassoued et al., 2017). The excellent activity and desirable magnetic recovery property render it useful in several applications (Garcia-Muñoz et al., 2020). For example, CoFe_2_O_4_ was effective for activating PMS to degrade atrazine (Li et al., 2018). CuFe_2_O_4_ and MnFe_2_O_4_ could be applied as catalysts of PS for acetaminophen and phenol removal (Stoia et al., 2017; Zhang et al., 2019). Further, in combination with PS, ZnFe_2_O_4_ exhibited good photocatalytic performance in the degradation of Orange II (Cai et al., 2016).

As mentioned, although certain ferrites have been applied to activate PS, the differences in the effectiveness of various ferrites in organics treatment have not been studied well. For example, metal species in a catalyst can critically impact the catalytic performance. Anipsitakis and Dionysiou (2004) reported that Fe^2+^ was the most efficient metal ion to activate H_2_O_2_, while Co^2+^ was the best for PMS activation and Ag^+^ showed the best results toward PS activation. The metal in M-site of MFe_2_O_4_ was known to be the main catalytic center for PS activation (Equation 1) (Li et al., 2017). However, the effect of different M-site metals in ferrite on PS activation to remove organics is not yet clear. Moreover, synthetic method is important for catalyst, which usually results in distinction on morphology, particle size, surface property, magnetism, etc., and thereby influences the catalytic performance (Kennaz et al., 2017; Zhang et al., 2018). Gupta and Garg (2017) found that compared with those prepared by coprecipitation and sol–gel methods, CuO/CeO_2_ synthesized by solution combustion method led to the maximum oxidation of organics and showed the minimum metal leaching in catalytic H_2_O_2_ system. Priyanka et al. (2019) found that modified TiO_2_ with lower band gap energy synthesized by sol–gel method had better mineralization of gray water in photocatalysis vs. those synthesized by ultrasonication and microwave methods. Ferrite can be also prepared by various methods including sol–gel, solvothermal, coprecipitation, and high-energy milling (Zhang et al., 2018). Hence, it is necessary to explore the effect of synthetic method of ferrite on organics removal in PS system.

(1) $$\begin{array}{l} M\left(\text{II}\right)+{\text{S}}_{2}{{\text{O}}_{8}}^{2-}\to M\left(\text{III}\right)+{\text{S}{\text{O}}_{4}}^{•-}+{\text{S}{\text{O}}_{4}}^{2-} \end{array}$$

In this work, the differences and causes in the catalytic performance of a series of MFe_2_O_4_ (M = Co, Cu, Mn, and Zn) were explored. Then, the characteristics and catalytic performances of ferrites synthesized by different methods, i.e., sol–gel, solvothermal, and coprecipitation, for activated PS process were investigated with CuFe_2_O_4_ as the representative. The efficacy of the catalyst was evaluated by applying it in the removal of two model refractory organics, a traditional dye pollutant [acid orange 7 (AO7)] and an emerging pharmaceutical pollutant (diclofenac). The main objectives were to (i) scrutinize the high-efficiency PS activator out and reveal the effect of M-site metal on the reactivity of ferrite and (ii) determine the effect of synthetic method on the performance of ferrite and find an ideal ferrite synthetic method for PS activation in organics treatment. The results can contribute to better understanding of the synthesis and application of ferrite and promote decontamination with activated PS process.

## Materials and Methods

### Materials

All chemicals used were of analytical grade. Ni(NO_3_)_2_·6H_2_O, Fe(NO_3_)_3_·9H_2_O, Zn(NO_3_)_2_·6H_2_O, NaOH, H_2_SO_4_ (95-98%), HCl (36-38%), C_2_H_6_O_2_ (ethylene glycol), and C_2_H_5_OH (ethanol) were purchased from Beijing Chemical Works, China. Cu(NO_3_)_2_·3H_2_O and Na_2_S_2_O_8_ were obtained from Tianjin Fuchen Chemical Reagents Factory, China. Co(NO_3_)_2_·6H_2_O and diclofenac sodium were purchased from Shanghai Macklin Biochemical Co. Ltd., China. Mn(NO_3_)_2_ (50% solution), C_6_H_8_O_7_·H_2_O (citric acid), C_2_H_3_NaO_2_·3H_2_O (NaAc), and Na_2_SO_4_ were obtained from Sinopharm Chemical Reagent Co. Ltd., China. AO7 was purchased from Tianjin Guangfu Fine Chemical Research Institute, China.

### Synthesis of Ferrite

A series of spinel ferrite MFe_2_O_4_ (M = Co, Cu, Mn, and Zn) were prepared by sol–gel method (Li et al., 2017) to investigate the effect of M-site metal on PS activation. Then, the three most common methods, sol–gel, solvothermal (Ueda Yamaguchi et al., 2016), and coprecipitation (Jaafarzadeh et al., 2017), were used to study the effect of synthetic method on the properties of ferrite obtained for PS activation, with CuFe_2_O_4_ as a representative. CuFe_2_O_4_ synthesized by sol–gel, solvothermal, and coprecipitation methods were denoted as CuFe_2_O_4_-SG, CuFe_2_O_4_-ST, and CuFe_2_O_4_-CP, respectively. The detailed synthetic procedures were described in the Supplementary Information.

### Characterization of Ferrite

X-ray powder diffraction (XRD) of ferrite was carried out on a Rigaku D/max-rc diffractometer using Cu *K*_α_ radiation. The morphology of ferrite was observed on a Hitachi S 4700 scanning electron microscope (SEM). N_2_ adsorption–desorption analysis was performed on a QuadraSorb Station 4 instrument. X-ray photoelectron spectra (XPS) were measured on a Thermo Fisher Scientific EscaLab 250Xi system with a monochromatic Al *K*_α_ source.

### Catalytic PS Oxidation Experiment

The typical experimental steps were as follows: known amounts of ferrite and PS solution were added simultaneously into a 20 mg/L organics solution (AO7 or diclofenac) under magnetic stirring. At known intervals, 3 ml solution was taken using a syringe and filtered through a 0.22 μm filter head. The concentration of the organics in the filtrate was analyzed to evaluate the efficacy of ferrite. The used ferrite was collected using a magnet, washed several times with ethanol and deionized water, and dried for the next run to investigate its reusability. The experiments were done in triplicate.

### Analytical Methods

The concentration of AO7 was determined by TU-1900 UV-visible spectrophotometer (Beijing Persee General Instrument Co., Ltd.) at a maximum absorbance wavelength of 484 nm. The concentration of diclofenac was determined by Ultimate 3000 high performance liquid chromatography (Thermo Fisher Scientific Inc.). The UV detection wavelength was 275 nm, and the mobile phase consisted of acetonitrile and 0.2% acetic acid solution at a volume ratio of 7:3.

The redox property of ferrite was evaluated by oxygen temperature-programmed oxidation (O_2_-TPO) and cyclic voltammetry (CV). O_2_-TPO was performed from 200 to 500°C at a rate of 10°C/min on a ChemBET Pulsar TPR/TPD instrument (Quantachrome Instruments Inc.) equipped with a thermal conductivity detector (TCD) to measure the change of gas composition. A 5% O_2_/He (vol.) gas mixture with a flow rate of 100 ml/min was used in the analysis. CV was conducted on a CHI 760E electrochemical workstation (Shanghai Chenhua instrument Co. Ltd.) with a foamed nickel working electrode, a platinum sheet counter electrode, and a saturated Ag/AgCl reference electrode. Before use, the foamed nickel electrode was dipped in the suspension of ferrite for 10 min to load the catalyst and then air-dried. A mixture of 0.1 mol/L Na_2_SO_4_ and 0.4 mmol/L PS was used as the electrolyte.

The surface hydroxyl group of ferrite was quantified by the saturated deprotonation method (Ren et al., 2015). In this method, 20 ml of a 0.05 mol/L NaOH solution dispersed with 0.12 g of ferrite was shaken for more than 4 h at 25°C. After separating the solid by filtration, the solution was titrated with a diluted HCl solution.

## Results and Discussion

### Catalytic Performance of Ferrites With Different M-Site Metals

To investigate the effect of M-site metal on the catalytic performance of spinel ferrite, a series of MFe_2_O_4_ (M = Co, Cu, Mn, and Zn) were synthesized by sol–gel method. XRD patterns presented in Supplementary Figure 1 confirmed the successful synthesis of the ferrite samples.

**Figure 1:**
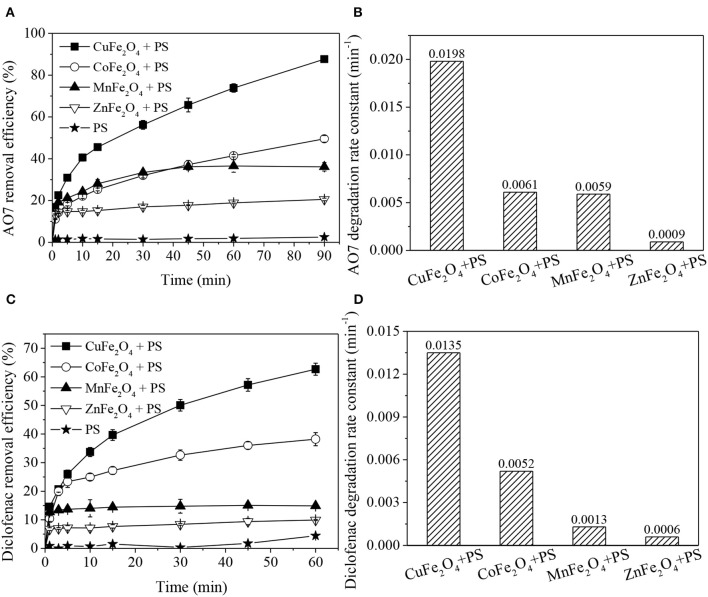
Organics removal and degradation rate constants in PS systems activated by ferrites with different M-site metals: **(A,B)** AO7 removal; **(C,D)** diclofenac removal. Conditions: [organics] = 20 mg/L, **(A,B)** catalyst dosage = 0.2 g/L, [PS] = 0.8 g/L, unadjusted pH = 6.5; **(C,D)** catalyst dosage = 0.6 g/L, [PS] = 0.1 g/L, pH = 5.

Figure 1 clearly shows that the removal efficiencies of organics in different MFe_2_O_4_ + PS systems were different, but all were higher than that of PS oxidation and MFe_2_O_4_ adsorption (Supplementary Figure 2). For both AO7 and diclofenac removal (Figures 1A,C), the catalytic performance of ferrite ranked as follows: CuFe_2_O_4_ > CoFe_2_O_4_ > MnFe_2_O_4_ > ZnFe_2_O_4_. The organics removal processes involved two stages: rapid adsorption-dominated stage (0-1 min) and catalytic degradation stage (> 1 min), which could be fitted by pseudo-first order reaction. The degradation rate constant (Figures 1B,D) was also significantly affected by M-site metal, and the trend was basically consistent with the aforementioned catalytic performance order of MFe_2_O_4_. In detail, CuFe_2_O_4_ presented the best and fastest catalytic performance in organics removal. Almost 87.6% AO7 was removed in PS solution coupled with CuFe_2_O_4_. In comparison, Yue et al. (2016) found that only 53.5% AO7 removal was obtained in Fe_3_O_4_ + PS system. Moreover, the diclofenac degradation rate constant of CuFe_2_O_4_ + PS system was about 3.5 times of that of thermally activated PS system at 60°C (Chen et al., 2016). The high removal of AO7 and diclofenac (which have different molecular structures: AO7 is an azo dye and diclofenac is a secondary aromatic amine drug) indicated that CuFe_2_O_4_ + PS could effectively remove multiple organic pollutants.

These results demonstrated that M-site metal indeed affected the catalytic performance of ferrite. Among the ferrites, CuFe_2_O_4_ was found to be the best activator of PS for organics removal.

### Redox Properties of Ferrites With Different M-Site Metals

According to above degradation experiments, it has been identified that ferrites with different M-site metals exhibit different catalytic performances. As is known, for PS activation by transition metal, the basic mechanism is chemical reduction of PS through electron transfer (Wacławek et al., 2017). Thus, the reducibility of catalyst is probably the vital factor affecting the effectiveness of PS activation system (Wang and Wang, 2018). Therefore, O_2_-TPO and CV were carried out to investigate the redox properties of ferrites with different M-site metals.

**Figure 2:**
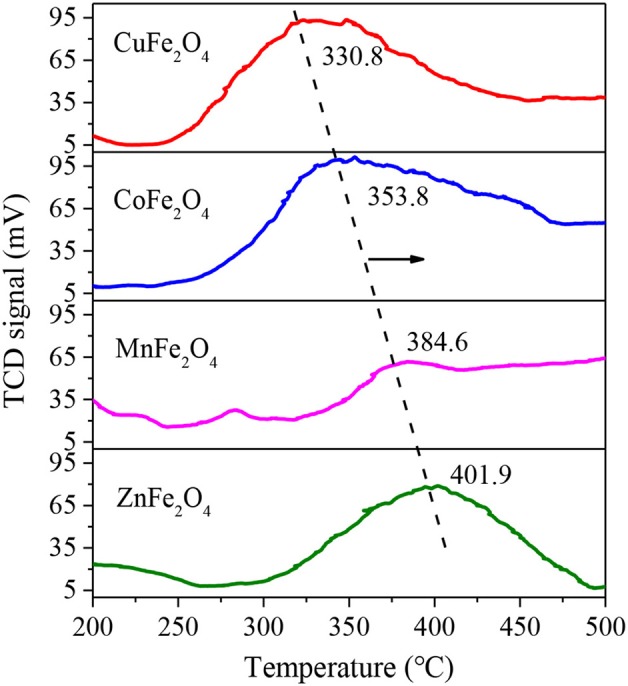
O_2_-TPO profiles of ferrites with different M-site metals.

Figure 2 shows the O_2_-TPO profiles of various MFe_2_O_4_. The temperature of O_2_ consumption surge is an important parameter for evaluating the ease of oxidation-state change of M-site metal ion. As shown, the four ferrites exhibited distinct peaks with temperature increasing from 200 to 500°C, implying the oxidation reaction occurrence of M^n+^ to M^(n+1)+^. The peak temperatures of CuFe_2_O_4_, CoFe_2_O_4_, MnFe_2_O_4_, and ZnFe_2_O_4_ gradually increased, at 330.8, 353.8, 384.6, and 401.9°C, respectively, which were similar to some other studies. For example, Wang et al. (2011) found that the initial oxidation of CoFe_2_O_4_ occurred at 350°C resulting from the oxidation of Co. Cihlar et al. (2017) reported that Mn^2+^ in binary oxide was mostly oxidized in the 350–500°C region. The lower oxidation temperature of MFe_2_O_4_, that was, the easier transition of the oxidation state of M-site metal ion accounted for its better performance in the activation of PS (Su et al., 2017).

**Figure 3:**
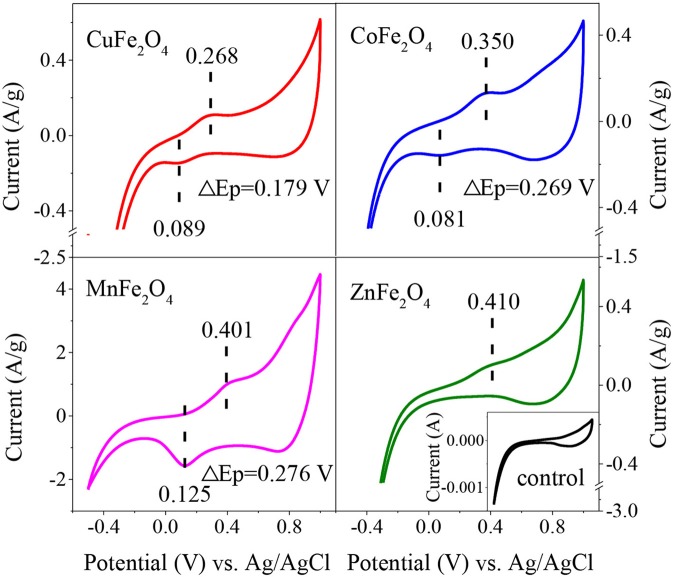
Cyclic voltammograms obtained on electrodes of ferrites with different M-site metals after 4th cycle. Scan rate = 50 mV/s, Scan range = −0.5-1 V.

To further reveal the redox properties of ferrites, CV curves of different MFe_2_O_4_ on electrodes were recorded (Figure 3). Except for the couple of redox peaks, the curves of all samples were identical in shape to the control curve, indicating that there was no interference of impossible peak in the solution. CuFe_2_O_4_ electrode exhibited a well-defined oxidation peak at 0.268 V, which was attributed to the Cu(II)/Cu(III) redox cycle. Likewise, the peaks at 0.350 and 0.401 V were assigned to the oxidation of Co(II) and Mn(II), respectively. ZnFe_2_O_4_ electrode gave an indistinct oxidation peak at 0.410 V. The lower potential of oxidation peak meant that it was easier for the catalyst to donate electrons, which was favorable for PS activation (Duan et al., 2018). Thus, the reducibility of the ferrites could be ranked as CuFe_2_O_4_ > CoFe_2_O_4_ > MnFe_2_O_4_ > ZnFe_2_O_4_. Moreover, there was no certain rule about the reduction peak positions of MFe_2_O_4_ electrodes. However, interestingly, for these ferrite electrodes, the trend of potential separation of redox peaks (ΔE_p_) was similar to the trend of oxidation peak potential. CuFe_2_O_4_ electrode displayed the lowest ΔE_p_ of 0.179 V. Meanwhile, the ΔE_p_ values of CoFe_2_O_4_ and MnFe_2_O_4_ electrodes were higher at 0.269 and 0.276 V, respectively. In the case of ZnFe_2_O_4_, it might need much more negative potential than that in the CV curve to make it accept electrons to generate reduction peak. The lower ΔE_p_ of an electrode indicated the stronger reversibility and more electron transfer of M^2+^/M^3+^ redox reaction (Bard et al., 1980), which were good for the catalytic reaction.

Therefore, from the above results, it can be concluded that M-site metal would affect the catalytic performance of MFe_2_O_4_ by affecting the reducibility, reversibility of M^2+^/M^3+^ redox couple, and electron transfer on the catalyst surface.

### Surface Oxygen Functional Groups of Ferrites With Different M-Site Metals

**Figure 4:**
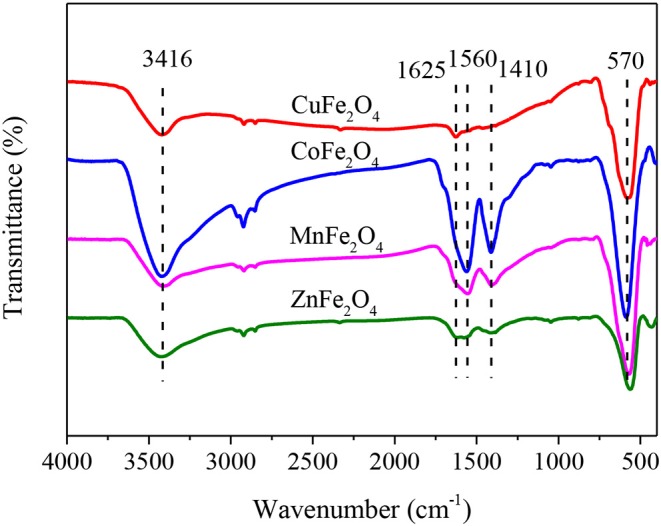
FTIR spectra of ferrites with different M-site metals.

The surface oxygen functional groups of a catalyst might participate in the activation of PS, and thereby affect the catalytic performance of the catalyst (Xiao et al., 2018). Therefore, ferrites with different M-site metals were characterized by FTIR spectra (Figure 4). The broad bands at about 3,416 cm^−1^ of all samples indicated the obvious presence of hydroxyl group (-OH) (Zhang et al., 2019). The peak at 1,625 cm^−1^ was due to the deformation vibration of water molecules in the interlayer (Parvas et al., 2014). The two adsorption bands at 1,560 and 1,410 cm^−1^ (COO- stretching) implied the residual of some citrates in the pores of ferrite. The peak at around 570 cm^−1^ was associated with the metal–oxygen bond (Zhao et al., 2018).

Among the observed functional groups, -OH should be of particular concern. It was reported that phenol removal was related to the surface hydroxyl concentration of TiO_2_ during catalytic ozonation (Song et al., 2010). Hydroxyl group on CuFe_2_O_4_ surface was found to be critical for radical generation in PMS activation (Guan et al., 2013). Therefore, surface -OH quantities of ferrites with different M-site metals were measured. As shown in Table 1, different M-site metals led to different -OH quantities. Unexpectedly, ferrite with good catalytic performance (e.g., CuFe_2_O_4_) did not have many surface -OH. This phenomenon was different from the finding of Ren et al. (2015) that MFe_2_O_4_ containing more surface -OH showed better catalytic performance for PMS. Ren et al. proposed that surface -OH was the main binding site for PMS (surface -OH of ferrite formed hydrogen bond with side -O-OH of PMS), and then PMS accepted electron from metal ion and its O-OH bond was broken to generate ${\text{S}{\text{O}}_{4}}^{•-}$. The results of this study showed that surface -OH was not crucial for the catalytic performance of MFe_2_O_4_ in PS system. The reason might be that PS activation involved a different process; ${\text{S}{\text{O}}_{4}}^{•-}$ in MFe_2_O_4_ + PS system was mainly generated from the fission of middle O-O bond of PS (Wang and Wang, 2018).

**Table 1:** Surface hydroxyl quantities of ferrites with different M-site metals.

**Ferrite**	**Surface hydroxyl quantity (mmol/g)**
CuFe_2_O_4_	0.75
CoFe_2_O_4_	3.13
MnFe_2_O_4_	2.58
ZnFe_2_O_4_	1.17

### Characterization of CuFe_2_O_4_ Synthesized by Different Methods

According to the above results, CuFe_2_O_4_ was selected as the representative ferrite to further explore the effect of synthetic method on the physicochemical property and catalytic performance of ferrite for PS activation.

#### XRD Analysis

**Figure 5:**
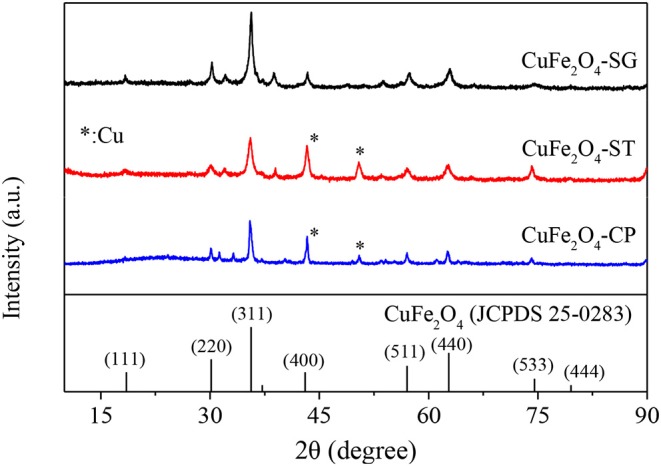
XRD patterns of CuFe_2_O_4_ synthesized by different methods.

The XRD patterns of CuFe_2_O_4_ synthesized by sol–gel, solvothermal, and coprecipitation methods (CuFe_2_O_4_-SG, CuFe_2_O_4_-ST, and CuFe_2_O_4_-CP) were shown in Figure 5. The major crystal phase of the samples was in agreement with typical spinel CuFe_2_O_4_ (JCPDS 25-0283), indicating that CuFe_2_O_4_ was indeed formed by all three methods. Moreover, no obvious impurity peak was found in the XRD pattern of CuFe_2_O_4_-SG, while two weak Cu (JCPDS 89-2838) diffraction peaks were observed in CuFe_2_O_4_-ST and CuFe_2_O_4_-CP. Meanwhile, the XRD peaks of CuFe_2_O_4_-SG were stronger and sharper. These results showed that ferrite prepared by sol–gel method was purer with better crystallinity than the samples prepared by the other two methods.

#### SEM Analysis

**Figure 6:**
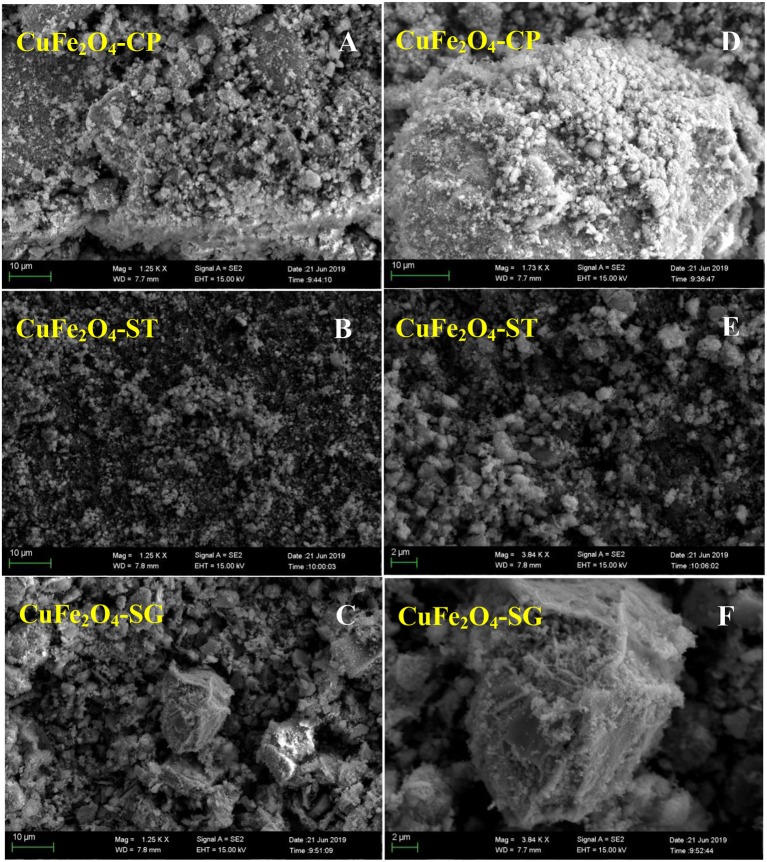
SEM images of CuFe_2_O_4_ synthesized by different methods: **(A–C)** 1250 times magnification; **(D)** 1730 times magnification; **(E,F)** 3840 times magnification.

The SEM images in Figure 6 show that the morphological structure of ferrite strongly depended on synthetic method. CuFe_2_O_4_-CP particles agglomerated into large and compact bulk forms, which might be caused by particle sintering that occurred during the calcination of coprecipitation precursor. This tended to reduce the contact area between the catalyst and other reactants, which was not conducive to pollutant removal (Xue et al., 2007). CuFe_2_O_4_-ST had a typical morphology of a solvothermal catalyst (Ueda Yamaguchi et al., 2016; Chen et al., 2017) with high dispersion, relatively uniform spherical-like shape, and minimum particle size, which could increase the external surface area. CuFe_2_O_4_-SG particles were found to be of moderate size and irregular shape. By the observation of enlarged SEM images of the three samples (Figures 6D–F), it is noteworthy that CuFe_2_O_4_-SG showed a spongy structure. Owing to the volatilization of citric acid, CuFe_2_O_4_-SG did not sinter as CuFe_2_O_4_-CP did, but had many discernible tiny pores. This porous structure was significantly advantageous in catalytic reaction because it could afford a large amount of reactive sites and enhance the reactant diffusion (Hou et al., 2018).

#### N_2_ Adsorption–Desorption Analysis

**Figure 7:**
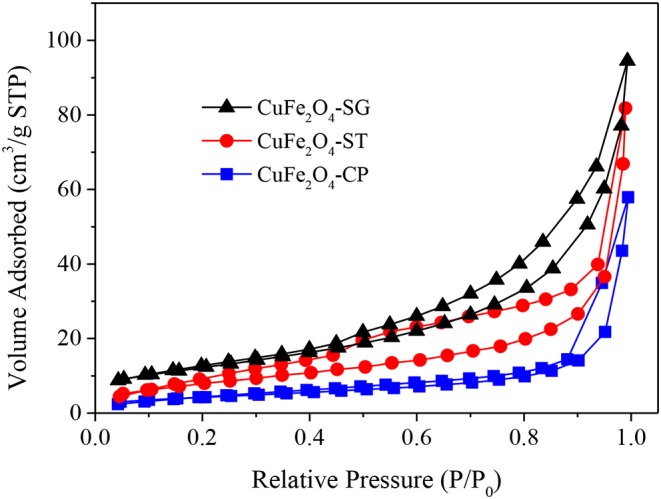
N_2_ adsorption-desorption isotherms of CuFe_2_O_4_ synthesized by different methods.

The N_2_ adsorption–desorption isotherms of the three CuFe_2_O_4_ were presented in Figure 7. The isotherms all belonged to Type IV curve with H3 hysteresis loop, which pointed to the disordered, lamellar mesoporous structure of the catalysts. Obviously, the adsorption capacity of CuFe_2_O_4_ ranked as follows: CuFe_2_O_4_-SG > CuFe_2_O_4_-ST > CuFe_2_O_4_-CP. Table 2 summarizes the basic structural parameters of the various as-synthesized CuFe_2_O_4_ samples. CuFe_2_O_4_-SG had the largest surface area and pore volume, followed by CuFe_2_O_4_-ST, and the values of CuFe_2_O_4_-CP were much lower than those of the former two, which was consistent with the SEM observation. Interestingly, the sequences of surface area and pore volume were completely consistent with the adsorption capacity of the prepared sample, but the mesopore size and particle size (observed in SEM images) did not follow this rule. These results indicated that sol–gel method endowed ferrite with a large surface area and pore volume, which played a pivotal role in the material's adsorption capacity, while the pore size and particle size were not the key factors affecting the adsorption capacity. When the adsorption capacity of ferrite was stronger, the amounts of pollutant and PS gathered were greater, and when the surface area was larger, the amount of active component exposed was greater, which were conducive to promoting the catalytic degradation of organics.

**Table 2:** Basic structural parameters of CuFe_2_O_4_ synthesized by different methods.

**Catalyst**	**Surface area (m^**2**^/g)**	**Pore volume (cm^**3**^/g)**	**Pore size (nm)**
CuFe_2_O_4_-SG	44	0.146	2.744
CuFe_2_O_4_-ST	30	0.130	1.964
CuFe_2_O_4_-CP	15	0.090	2.775

#### XPS Analysis

**Figure 8:**
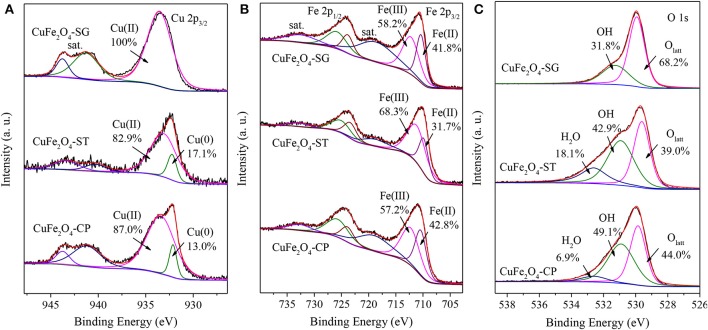
XPS spectra of CuFe_2_O_4_ synthesized by different methods: **(A)** Cu 2p, **(B)** Fe 2p, and **(C)** O 1s.

Figure 8 presents the surface elements chemical state of CuFe_2_O_4_ synthesized by different methods. As shown in Figure 8A, all samples yielded Cu(II) 2p_3/2_ peak at around 933.4 eV along with two satellite peaks at 941.3 and 943.8 eV (Lei et al., 2015). However, the surface of CuFe_2_O_4_-SG consisted uniquely of Cu(II); the surfaces of CuFe_2_O_4_-ST and CuFe_2_O_4_-CP also contained a small proportion of Cu(0) (the peak at 932.2 eV) (Li et al., 2019). The calculation of peak area showed that CuFe_2_O_4_-ST contained more surface Cu(0) than CuFe_2_O_4_-CP contained, which was in agreement with the diffraction peak intensities in the XRD patterns.

Figure 8B shows that Fe(III) (2p_3/2_ 712.2 eV, 2p_1/2_ 725.9 eV) and Fe(II) (2p_3/2_ 710.4 eV, 2p_1/2_ 723.8 eV) coexisted on the surface of the three CuFe_2_O_4_ samples (Li et al., 2019; Zhang et al., 2019). Obviously, the proportion of Fe(II) in CuFe_2_O_4_-SG and CuFe_2_O_4_-CP was much higher than that in CuFe_2_O_4_-ST. These results were favorable for organics removal because Fe was also an electron donor for PS activation (Zhang et al., 2019).

The O 1s spectra of CuFe_2_O_4_ synthesized by the three methods were shown in Figure 8C. All three samples showed peaks of lattice oxygen (O_latt_, 529.9 eV) and OH adsorbed on the surface (531.3 eV) (Li et al., 2017). The highest proportion of O_latt_ in CuFe_2_O_4_-SG suggested the good crystal structure obtained by sol–gel method. The spectra of CuFe_2_O_4_-ST and CuFe_2_O_4_-CP contained another peak at 532.6 eV ascribed to adsorbed H_2_O (Zeng et al., 2017). The high proportion of adsorbed OH and H_2_O on the surface indicated the strong hydroxylation of CuFe_2_O_4_ during coprecipitation and solvothermal processes (Wang et al., 2019).

### Catalytic Performance of CuFe_2_O_4_ Synthesized by Different Methods

**Figure 9:**
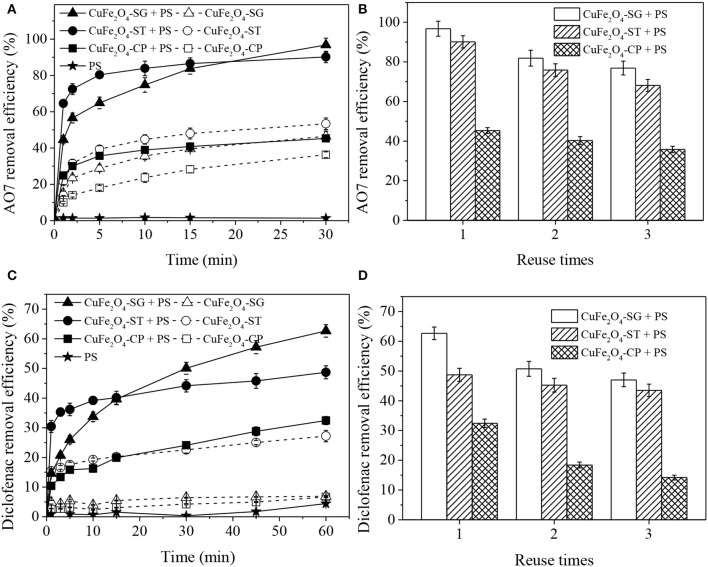
Organics removal in different CuFe_2_O_4_ activated PS systems and reusability of CuFe_2_O_4_ synthesized by different methods: **(A,B)** AO7 removal; **(C,D)** diclofenac removal. Conditions: [organics] = 20 mg/L, [catalyst] = 0.6 g/L, **(A,B)** [PS] = 0.8 g/L, unadjusted pH = 6.5; **(C,D)** [PS] = 0.1 g/L, pH = 5.

Figure 9 displays AO7 and diclofenac removal in different CuFe_2_O_4_ + PS systems. As seen, the effectiveness and reusability of the catalyst were significantly influenced by synthetic method.

Figure 9A shows that CuFe_2_O_4_-SG had the best PS catalytic performance. AO7 removal efficiency in CuFe_2_O_4_-SG + PS, CuFe_2_O_4_-ST + PS, and CuFe_2_O_4_-CP + PS systems were 96.8, 90.1, and 45.3%, respectively. In contrast, only 46.5, 53.4, and 36.3% of AO7 were adsorbed by the corresponding catalyst alone. In the early stage of reaction, the relatively large external surface area of the catalyst enabled CuFe_2_O_4_-ST to activate PS to remove more AO7. However, as the reaction proceeded, PS and AO7 gradually diffused into the pores of CuFe_2_O_4_-SG and reacted on the abundant active sites, ultimately leading to a better removal of AO7. Meanwhile, the corrosion and dissolution of the metallic Cu impurity observed on CuFe_2_O_4_-ST reduced the content of active metal and led to a gradual loss of activation ability of CuFe_2_O_4_-ST (Li et al., 2019). As for CuFe_2_O_4_-CP, its adsorption and catalytic capacity were greatly hindered owing to the serious sintering. Moreover, the reusability of CuFe_2_O_4_ synthesized by different methods was also found to be different (Figure 9B). After three runs, the decrease of AO7 removal in CuFe_2_O_4_-SG + PS system (19.9%) was lower than that in CuFe_2_O_4_-ST + PS system (22.0%). AO7 removal efficiency in CuFe_2_O_4_-SG + PS, CuFe_2_O_4_-ST + PS and CuFe_2_O_4_-CP + PS systems became 76.9, 68.1, and 35.8%, respectively. The lower decreasing trend of activity and higher pollutant removal efficiency after repeated uses suggested that CuFe_2_O_4_-SG had a good reusability. The removal of diclofenac in different CuFe_2_O_4_ + PS systems (Figures 9C,D) was similar to that of AO7. Once again, CuFe_2_O_4_-SG showed the best performance, followed by CuFe_2_O_4_-ST and then CuFe_2_O_4_-CP.

These results demonstrated that synthetic method would influence the catalytic performance of ferrite from morphological structure, surface area, and element chemical state. Sol–gel method was the ideal one to synthesize ferrite applicable in activated PS process.

## Conclusions

M-site metal and synthetic method significantly influenced the catalytic performance and physicochemical property of spinel ferrite. The sequence of the effectiveness of ferrite-activated PS system for organics removal was CuFe_2_O_4_ > CoFe_2_O_4_ > MnFe_2_O_4_ > ZnFe_2_O_4_. The high catalytic performance of MFe_2_O_4_ resulted from its good reducibility, strong reversibility of M^2+^/M^3+^ redox couple, and active electron transfer on the surface, which were affected by M-site metal. Surface -OH was not crucial for the catalytic performance of MFe_2_O_4_ in PS system. Moreover, sol–gel method was found to be the ideal one to synthesize ferrite to effectively activate PS for organics removal. The as-prepared ferrite had good purity, porous structure, large surface area, and favorable element chemical states, leading to superior catalytic performance and reusability compared with those prepared by solvothermal and coprecipitation methods. The results served as a reference for screening ferrite and promoting PS activation.

## Data Availability Statement

All datasets generated for this study are included in the article/Supplementary Material.

## Author Contributions

GX, SK, GZ, and NZ designed the experiments. GX, SK, QL, and HD performed the experiments. GX, SK, and GZ wrote the paper. GX, SK, QL, GZ, NZ, HD, and LN discussed the results and analyzed the data.

### Conflict of Interest

The authors declare that the research was conducted in the absence of any commercial or financial relationships that could be construed as a potential conflict of interest.
