# Supplementary material for: Synthesis of Spinel Ferrite MFe2O4 (M = Co, Cu, Mn, and Zn) for Persulfate Activation to Remove Aqueous Organics: Effects of M-Site Metal and Synthetic Method
Source: Front Chem. 2020 Mar 24;8:177. doi: 10.3389/fchem.2020.00177 (PMC7105867; doi:10.3389/fchem.2020.00177)
Supplement: Supplementary file 1 [file Data_Sheet_1.docx]

***Supplementary Material***

1. **Detailed procedures of ferrite MFe_2_O_4_ synthetic methods**

Sol-gel (SG) : an aqueous solution of M(NO_3_)_2_ and Fe(NO_3_)_3_ (M:Fe molar ratio = 1:2) was stirred at 60 °C for 2 h on magnetic agitator, then citric acid with molar mass which was almost equal to the sum of cations’ moles was added. After stirring for another 2 h at 60 °C, the solution was placed in water bath (90 °C) to evaporate water. The resultant gel was calcined at 400 °C for 2 h in tube furnace with 200 mL/min N_2_.

Solvothermal (ST): 4.75 mmol M(NO_3_)_2_·and 9.5 mmol Fe(NO_3_)_3_ were dispersed in 75 mL ethylene glycol with 30 min stirring and 30 min ultrasonication of 0.3 W/mL. Then, 7.5 g NaAc was added, followed by 1 h stirring. The mixture was transferred into a 100 mL Teflon-lined stainless steel autoclave and heated at 200 °C for 10 h. The obtained solid was washed by ethanol and deionized water several times and dried at 60 °C.

Co-precipitation (CP): an aqueous solution of M(NO_3_)_2_ and Fe(NO_3_)_3_ (M:Fe molar ratio = 1:2) was stirred at room temperature for 30min. Latter, 5 mol/L NaOH was added dropwise until the solution pH reached 12, followed by another 30 min stirring at 80 °C. Then, the mixture was aged in water bath (80 °C) for 3 h. After cooling, the precipitate was separated by magnet, washed by deionized water several times and dried at 80 °C. The obtained solid was calcined at 400 °C for 2 h in tube furnace with 200 mL/min N_2_.

1. **Supplementary Figures**

**Supplementary Figure 1.** XRD patterns of ferrites with different M-site metals.

**Supplementary Figure 2.** **(A)** AO7 and **(B)** diclofenac removal by MFe_2_O_4_ adsorption and PS oxidation. Conditions: [organics] = 20 mg/L, **(A)** MFe_2_O_4_ dosage = 0.2 g/L, [PS] = 0.8 g/L, unadjusted pH = 6.5; **(B)** MFe_2_O_4_ dosage = 0.6 g/L, [PS] = 0.1 g/L, pH = 5.
